# Parental perspectives on the awareness and delivery of preconception care

**DOI:** 10.1186/s12884-017-1531-1

**Published:** 2017-09-26

**Authors:** M. Poels, M. P. H. Koster, A. Franx, H. F. van Stel

**Affiliations:** 1 Department of Obstetrics and Gynecology, University Medical Center Utrecht, P.O. Box 85090, 3508 AB Utrecht, the Netherlands; 2 Department of Healthcare Innovation and Evaluation, Julius Center for Health Sciences and Primary Care, University Medical Center Utrecht, P.O. Box 85500, 3508 GA Utrecht, the Netherlands; 3 Present address: Department of Obstetrics and Gynecology, Erasmus MC, P.O. Box 2040, 3000 CA Rotterdam, the Netherlands

**Keywords:** Preconception care, Women’s experiences, Maternal health, Access to health care, Reproductive health, Midwifery, Qualitative research

## Abstract

**Background:**

The attention for Preconception Care (PCC) has grown substantially in recent years, yet the implementation of PCC appears challenging as uptake rates remain low. The objective of this study was to assess parental perspectives on how PCC should be provided.

**Methods:**

Recruitment of participants took place among couples who received antenatal care at a Dutch community midwifery practice. Between June and September 2014, five focus group sessions were held with 29 women and one focus group session with 5 men. Thematic analysis was conducted using NVivo 10 software.

**Results:**

Participants were generally unfamiliar with the concept of PCC. It was proposed to raise awareness by means of a promotional campaign, stipulating that PCC is suited for every couple with a (future) child wish. Suggestions were made to display marketing materials in both formal and informal (local community) settings. Addressing existing social networks and raising social dialogue was expected to be most efficient. It was recommended to make PCC more accessible by offering multiple forms and to involve male partners. Opportunistic offering PCC by healthcare providers was considered more acceptable when the subject was deliberately raised, for example while discussing contraceptives, lifestyle risks or drug prescriptions. GP’s or midwifes were regarded the most suitable PCC providers, however provider characteristics such as experience, empathy and communication skills were considered more important.

**Conclusions:**

This study showed that from the parental perspective it is recommended to address every couple with a (future) child wish by means of enlarging the awareness and accessibility of PCC. In order to enlarge the awareness, it is recommended to address social networks, to raise the social dialogue and to conduct promotional campaigns regarding PCC. In order to improve the accessibility of PCC, it was suggested to simultaneously offer multiple forms: group sessions, individual consultations, walk-in-hours and online sessions, and to involve male partners.

## Background

Today, it is internationally recognized that the organization of obstetric care should increasingly focus on the preconception period to prevent adverse pregnancy outcomes, since this is a critical period in which organogenesis occurs [1, 2]. Previous research has shown that almost all couples who are trying to conceive have at least one risk factor for which individual counseling by a healthcare provider is indicated [3–5]. Preconception care (PCC) has the potential to timely address those risk factors to positively affect maternal and child health [6, 7]. PCC has previously been defined as “a set of interventions that aim to identify and modify medical, behavioral and social risks to a woman’s health or pregnancy outcome through prevention and management” [6–8]. The three key elements of PCC are risk prevention, health promotion and interventions [9]. There are several ways of providing PCC, varying from individual PCC counseling, group information sessions, and online education to national folic acid fortification programs [6, 9–11]. Individual PCC counseling starts with screening of the presence of the couple’s risk factors, including lifestyle, infectious diseases, genetic history, immunization status, chronic illness, medication use, mental health and working conditions. Subsequently, several advices can be provided including daily folic acid and vitamin supplementation, obtaining a healthy weight, quit smoking and drinking and altering teratogenic medications and avoidance of exposure to chemicals. Some of these advices may call for targeted interventions, for example a weight-reduction plan or smoking cessation program [3, 7, 9, 11, 12].

The attention for PCC has grown substantially in recent years, yet in most countries it has not become part of routine practice and the use of PCC among prospective parents remains low [13–19]. One of the major challenges regarding PCC is to identify how it can best be delivered in order to improve its uptake [10]. Few studies have been conducted on this matter and mostly primarily aimed at the views of healthcare providers. Research on parental attitudes is often bound to women’s motives for (not) participating in PCC and shows that perceived sufficient knowledge, lack of awareness and planning issues inhibit the uptake of PCC [8, 16, 20, 21]. Yet, the literature regarding parental perspectives on the delivery of PCC is scant and almost always only performed among women. More insight into both women’s and men’s experiences and needs for the delivery of PCC is essential for the successful implementation of PCC [22, 23]. Therefore, the objective of this study was to assess parental perspectives on how PCC should be provided.

## Methods

### Study participants

Participants were recruited from the only community midwifery practice in the suburban municipality Zeist, the Netherlands. Recruitment took place among respondents of a questionnaire study (*n* = 283) regarding PCC, which was held from February–April 2014. Those were women who gave birth to a live-born between January and September 2013. After completion of the questionnaire, 105 women (37.1%) gave consent to be invited for a focus group regarding PCC. We allocated these women to five recruitment pools, based on their medical risk, educational level and ethnicity, since we aimed to conduct stratified focus groups. Women were invited to participate in a focus group at a fixed date and time by telephone. Non-responders were called back twice and then sent an invitation by e-mail. Participating women were also asked to recruit their male partners for participation in a focus group with men. When consent was obtained, the male partner was included.

### Data collection

The focus group sessions were guided by a trained moderator (MP) and an assistant (HvS, WK), experienced in conducting focus group sessions. To maintain consistency, the focus groups with women were conducted by the same female moderator. A male moderator with vast experience in moderating male discussion groups, conducted the focus group with men. None of the participants had former involvement with the researchers. The focus groups were held at the office of the community midwifery practice at a central location in Zeist. The sessions were conducted in the Dutch language, which was mastered by all participants. For the purpose of publication, quotes were translated into the English language by two researchers (MP, HvS). Two weeks prior to the session, participants received an information letter containing information on practical issues, the study project, objectives and concept of PCC. Preconception care was defined as: “*all kinds of care and information you receive, before you conceive, to prepare your pregnancy as good as possible*” This could include searching for information on the Internet, a preconception care consult or conversation with your general physician, midwife, other health professional or someone familiar. This could concern your pregnancy wish, nutrition, smoking, alcohol use, chronic or hereditary illness or medication use. Before commencement of the focus group sessions, the objectives, format, anonymity and the concept of PCC were explained again verbally. The focus group sessions took 90 min and consisted of two main parts: 1) motives to participate in PCC and 2) perspectives and needs for the delivery of PCC. Both parts were structured by 3–4 main questions and conducted in a conversation-like manner. All participants were parents at the time of the sessions and were asked to answer questions in retrospect regarding the pregnancy of their latest child. This study has been approved by the Medical Ethical Review Board of the UMC Utrecht (protocol no. 13–475) and all participants gave informed consent to participate.

### Data analysis

The focus group sessions were audiotaped and transcribed verbatim. NVivo 10 software was used to extract and analyze the data. Thematic analysis was employed to identify key issues and themes [24]. One author (MP) developed an initial coding frame by reading and rereading the extracted findings. Additional topics that emerged from reviewing the transcripts were used to refine the initial coding frame. Extraction and coding was verified by a second author (HvS). Discrepancies were discussed until consensus was attained and both authors agreed on final analyses.

## Results

### Study participants

**Fig. 1:**
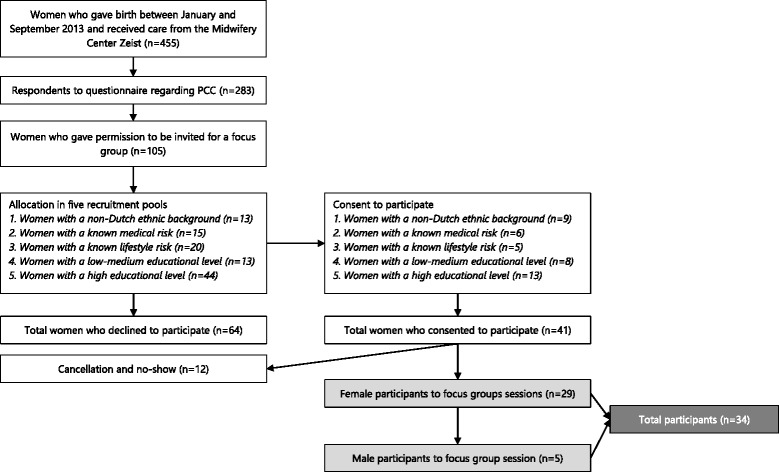
Recruitment of study participants

Six focus group sessions were held between June and September 2014 with 29 women and 5 men (Fig. 1). Non-responders were either not available at the given date and time to participate in a focus group or did not respond to the invitation. The characteristics of the participants are presented in Table 1. All participants were residents of the municipality of Zeist. Thirty-one participants were Dutch and three female participants originated from non-Western countries, i.e. China, Turkey and Afghanistan respectively. Ten women had a higher risk for pregnancy complications preceding their latest pregnancy, which concerned either a lifestyle risk (smoking, using alcohol or having a BMI >30), a medical risk (prior pregnancy complications, chronic/hereditary illness or medication use), or both. One man had a hereditary chronic disease.

**Table 1:** Characteristics of study participants

ID	Ethnicity	Educ. Level	# children	Known risk factor
*Focus group 1. Women with a non-Dutch ethnic background*				
F1P1	Chinese	High	2	
F1P2	Turkish	High	1	
F1P3	Afghan	Medium	4	
*Focus group 2. Women with a known medical risk*				
F2P1	Dutch	High	3	prior pregnancy complications
F2P2	Dutch	Low	2	chronic illness
F2P3	Dutch	High	2	prior pregnancy complications, preconceptional smoking and BMI >30
F2P4	Dutch	High	3	prior pregnancy complications
F2P5	Dutch	High	1	chronic illness/prior pregnancy complications
*Focus group 3. Women with a known lifestyle risk*				
F3P1	Dutch	High	1	BMI >30 and chronic illness
F3P2	Dutch	High	1	alcohol use during pregnancy and chronic illness
F3P3	Dutch	High	1	alcohol use during pregnancy
F3P4	Dutch	Medium	1	preconceptional smoking
F3P5	Dutch	High	1	BMI >30
*Focus group 4. Women with a low-medium educational level*				
F4P1	Dutch	Medium	3	
F4P2	Dutch	Medium	2	
F4P3	Dutch	Medium	1	
F4P4	Dutch	Medium	2	
*Focus group 5. Women with a high educational level*				
F5P1	Dutch	High	3	
F5P2	Dutch	High	1	
F5P3	Dutch	High	1	
F5P4	Dutch	High	3	
F5P5	Dutch	High	2	
F5P6	Dutch	High	1	
F5P7	Dutch	High	2	
F5P8	Dutch/Moroccan	High	2	
F5P9	Dutch	High	2	
F5P10	Dutch	High	2	
F5P11	Dutch	High	1	
F5P12	Dutch	High	1	
*Focus group 6. Men*				
F6P1	Dutch	High	2	
F6P2	Dutch	Medium	1	
F6P3	Dutch	Medium	3	
F6P4	Dutch/Asian	High	2	
F6P5	Dutch	High	2	hereditary chronic illness

### The concept of “preconception care”

In all focus group sessions, both women and men were generally unfamiliar with PCC. The common notion was that couples have to try to conceive for over 1 year before they are allowed to consult a healthcare provider. Many participants had no clear idea of the concept of PCC and experienced difficulties understanding the phrase “Preconception Care”. Almost all participants considered PCC suitable in the presence of certain risk factors (fertility problems, previous pregnancy complications, higher age, chronic illness, hereditary diseases and lifestyle issues, such as being overweight, smoking, alcohol or drug use), as these factors may require professional advice. Envisaging the relevance of PCC for all couples contemplating pregnancy was not as straightforward. Being young, healthy and already having children diminished the perceived need for PCC. In four focus group sessions, it was proposed to raise awareness by promoting PCC as a common type of care for every couple with a (future) child wish, stipulating that it is not necessary to wait until fertility issues arise (Quotes in Table 2).

**Table 2:** Quotes from the focus group sessions

*The Concept of Preconception Care*
F4, P4, female. “Well, I was a bit sober about it. I thought I’m only trying for 6 months now, so there’s no need to visit a midwife yet. But indeed, when it gets promoted that you can visit them from the moment you’re seriously considering [pregnancy], then the step to go would be easier. I would’ve attended [PCC].”
F4, P2, female. “Often people think you have to try for yourself first. I think that when it [PCC] gets more accessible, people will attend sooner. People make it too big. I would have probably attended as well.”
*The value of Preconception Care*
F4, P1, female. “I always get kind of afraid from these blogs and forums. It’s all kind of hysterical. One person tells this and the other person tells that, I never believe in those things and prefer to get my information from professionals.”
F5, P4, female. “I would value the customized information. On the Internet you read general things and advices. I would like to get specific information for my own situation, for my uncertainties and feeling of, well, fear. Customized care.”
F6, P2, male. “You can find so many contradictory information on the Internet. When you can speak to someone face to face who has the expertise it is reliable. Especially when you get into a phase in which becoming pregnant doesn’t succeed, it could be nice to talk with someone like that. Or in case you have doubts, use medication or smoke being the male partner. That you can ask questions.”
F6, P3, male. “I think that men are more hesitant to go. Maybe they think: well if my wife knows, I will hear it from her. They might be less enthusiastic to attend, while it is very important that men do.”
*Intrinsic motivation and responsibility for PCC*
F5, P1, female. “That you can deliver a healthy child, in a way that is healthy for you as a woman.”
F1, P2, female. “You are responsible. Eventually you knowingly made the choice for a baby. It doesn’t happen suddenly. It is a living being in your belly and personally I think that you have to behave responsibly.”
F2, P5, female. “I am convinced that a health professional has a relationship with his patient that goes beyond the patient’s demands. You should strive for optimal care which sometimes includes giving unsolicited advice.”

### The value of preconception care

Most women mentioned the Internet as their primary source of information regarding preconception health and were able to retrieve reliable preconception health information online. Especially higher educated women found themselves capable of segregating objective from subjective information online. Some lower to middle educated women acknowledged the presence of many social platforms and unreliable websites that have the potential to induce anxiety. Yet, in the majority of the focus group sessions both men and women expressed that a preconception consult with a healthcare provider could be valuable, as professional information was considered reliable and up-to-date. Moreover, personal contact was appreciated as it allows for dialogue, asking questions and suited applicable information. Some participants even believed that the prospect of PCC could relieve stress. Men especially valued the possibility of support and guidance and the opportunity to discuss any concerns or doubts. They felt to be branched at the sideline as women are considered the center of attention during pregnancy. Both men and women felt that men should be more involved in the pregnancy process, including the preconception period (Quotes in Table 2).

### Intrinsic motivation and responsibility for PCC

Women expressed that their intrinsic motivators to use PCC are pregnancy preparation, having a healthy pregnancy, becoming a healthy mother and having a healthy baby. Optimizing the chances of one’s future child was the major reason to attend a preconception care consult, as it was mentioned in four focus group sessions. Moreover, women stipulated that they felt responsible for their pregnancy and their choices on lifestyle matters, to resist temptations such as smoking and alcohol use and to change their eating habits during pregnancy. A few women believed that health professionals have a responsibility as well to inform their patients regarding their future pregnancy, especially when this professional is aware of the presence of (lifestyle) risks (Quotes in Table 2).

### Natural process & privacy

During focus group sessions, it was expressed that pregnancies are not always deliberately planned. Conceiving sooner than expected hindered some women to seek out PCC. Both men and women expressed having a planning attitude of “let things happen” and “we will see”. Men acknowledged that it is in their nature to react rather than to prepare. Apart from this, in one focus group session it was suggested that PCC could induce stress, make pregnancy plans too real and thereby interfere with the natural process of becoming pregnant. By contrast, one woman valued PCC as it could actually prevent her from going into the medical realm. In four focus group sessions, women emphasized that their pregnancy wish was an intimate decision between them and their partners. They were reluctant to share their plans with other people, including health professionals. This wish for secrecy was reflected in three focus group session in which women’s fear and shame was discussed to be spotted by relatives or acquaintances in the waiting room of a midwifery practice or gynecologist office. Yet, the waiting room of a GP was considered less intimidating, as one could visit the GP for a variety of other reasons than pregnancy plans. Still, women admitted to prefer the privacy of their own home to search for information. Therefore, it was suggested to offer PCC in alternative forms through the use of e-health. To guarantee anonymity, one woman suggested PCC to be offered in another municipality (Quotes in Table 3).

**Table 3:** Quotes from the focus group sessions

*Natural process & privacy*
F6, P4, male. “I looked at it quite loosely. We, my wife and I, just decided on one night while sitting at the couch “lets become parents”. That was it. My wife got pregnant very quickly and we didn’t look at the Internet at all. We just let it happen. That was just it. We didn’t want to let ourselves drive crazy.”
F2, P5, female. “I want to let it go naturally. It is a natural process. It is a choice in your relation, in your life. If I’m so occupied with it in advance, it might become a hyperfocus and the spontaneity gets lost.”
F5, P9, female. “The wish for children is really close. Then you think of your partner, but no one else. I think that such a private moment, the pre-phase, that I don’t have to discuss this with an outsider.”
*PCC in social context*
F1, P3, female. “When I know that a girlfriend has a pregnancy wish, I explain to her what things she should be aware of. I emphasize that she can do tests to make sure she will have a healthy child. I tell this to everyone, every friend or women that I see in my surroundings of family and friends.”
F5, P12, female. “Well, you could make it a standard kind of care, so your female friends don’t laugh at you when you visit such an office hour but think “wow maybe I should consider that too”. You could make it standard, within the whole package of pregnancy, labour and child welfare.”
F1, P2, female. “I have talked about this a lot with female friends when I wasn’t pregnant yet and when I became pregnant. Questions like: are you pregnant yet? How do you do it? Did you succeed or not? Those kind of conversations.”
*The acceptability of opportunistic PCC*
F3, P1, female. “I think that it’s hard for healthcare providers to estimate what the right timing is and how to address the issue, because it can be sensitive. That makes this more difficult compared to care during the pregnancy.”
F3, P5, female. “You go the GP with a reason, for example sinusitis and you get antibiotics. The GP could carefully raise: “I don’t know if you are considering children, but know that you should pay attention with this medication or do you have any other questions about that”. That way you create an entry.”
F6, P3, male. “I find it appropriate to raise this subject when side-effect from medication or overweight is discussed. Then I would accept this more compared to raising it out of the blue after a treatment.”

### PCC in social context

Participants felt that PCC should become just as self-evident as prenatal care. In four focus group sessions, it was urged for PCC to become a more common, usual, and standard type of care for every couple with a (future) child wish. Preferably, attending PCC becomes an obvious step, like midwifery visits once pregnancy occurs. To accomplish this, raising the social dialogue for PCC was considered useful. In two focus group sessions, participants expressed that word-of-mouth marketing would be a good way to enlarge awareness. Although privacy was identified as an interfering factor to openly discuss current pregnancy attempts, women explained that it is common to discuss issues regarding pregnancy and childbirth with relatives, female friends and wider social circles. In one focus group session it was suggested that, when women start to point out the relevance and need for PCC to each other, the responsibility for PCC could be shared socially. In all focus group sessions, it was proposed to send promotional messages through formal and informal channels and to use existing social networks and community settings. In addition to formal and healthcare related settings, suggestions were made to display marketing materials in schools, community centers, day care facilities, city hall, public places, mosques, churches, grocery stores, the library, the cinema, gyms, the swimming pool, etc. (Quotes in Table 3).

### The acceptability of opportunistic PCC

There were contrasting views among both men and women regarding the acceptability of health professionals opportunistically offering PCC. Approximately half of the participants felt the inquiry about pregnancy plans to be meddlesome, confronting or even painful. The other half of the participants had the opinion that more information would not be harmful and that it is up to a couple to decide to act upon this information. However, it was acknowledged that it may be hard for professionals to estimate whether future pregnancy plans are in order. Commonly, opportunistically offering PCC by healthcare providers was considered more acceptable when the subject was deliberately raised, for example while discussing contraceptives, pap smears, chronic illness, lifestyle risks or drug prescriptions. Men clearly expressed not to appreciate an “out of the blue” question regarding their child wish (Quotes in Table 3).

### Practical issues & forms of PCC

In all focus group sessions, it was recommended to make PCC more accessible by offering multiple forms: group sessions, individual consultations, walk-in-hours and online sessions. Participants advised to make PCC available for everyone, addressing a wider public, while offering customized care. During one focus group session, it came across that PCC was expected to be time-consuming, making it difficult to attend in women’s already busy lives. Additionally, some participants felt reluctant to bother health professionals with a high workload with questions regarding PCC. Walk-in-hours, telephone consultation, e-mail and Skype were proposed as less time-consuming alternatives for PCC (Quotes in Table 4).

**Table 4:** Quotes from the focus group sessions

*Practical issues & forms of PCC*
F4, P3, female. “You could also do both consultations and group sessions. Some people prefer it the one way, other people the other way. Some people might prefer the anonymity of a group, without any obligations, without the need to make an appointment. Then they can already get some information and if they have any specific questions they can make an appointment.”
F5, P4, female. “I think you should make it accessible and available for everyone and then offer customized care for the patient in question.”
*Provider characteristics*
F4, P1, female. “It think that the GP should take the lead. In this regard, the GP is most familiar and knows about history and perhaps about smoking, drugs. The GP refers for other health issues as well, so I think it‘s logical. He could give advice, for example to visit a group session.”
F5, P9, female. “I could image that when you have tough questions, that it has to do with feeling as well. Maybe more than you sometimes think. The conversations I had with midwives were of much more value because they touched me by their experience. When it comes to behavior or lifestyle you have to make choices, then it is an advantage when a professional really touches you.”
F1, P2, female. “I think people listen better to a GP or midwife then to someone they know from their neighborhood. They come through professionally and could explain the importance in a different manner.”

### Provider characteristics

The majority of participants regarded GP’s or midwives the most suitable PCC providers, while gynecologists, dieticians, physiotherapists, social workers and pharmacists were less frequently mentioned. The GP was denoted as the first health professional by whom PCC could be discussed confidently, as GP’s were considered most familiar, knowing about the family situation and the presence of risks factors, diseases or medication. By contrast, some participants felt more at ease discussing their pregnancy plans with an outsider, a professional who is unaware of their personal situation. Above all, individual provider characteristics such as experience, empathy and communication skills were regarded most important. In one focus group session, participants stressed that messages will not come across by information alone, but that cogency is needed to accomplish lifestyle changes (Quotes in Table 4).

### Differences in subgroups

The highly educated subgroup was most explicit in the opinion that PCC is suited for other people with risks. Moreover, participants from this subgroup experienced a lesser need for PCC as they found themselves very capable of retrieving the right preconception health information. From all subgroups, the non-western ethnicity subgroup valued the involvement of the social network the most, as they elaborated on social dialogue, word-of-mouth marketing and social responsibility. The male subgroup discussion did not yield any new themes other than topics men would like to discuss during a preconception care consult, such as how to support their female partner during pregnancy.

## Discussion

This study showed that (future) parents recommend to address every couple with a (future) child wish by means of enlarging the awareness and accessibility of PCC. In order to enlarge the awareness, it was recommended to address existing social networks, to raise the social dialogue, and to conduct promotional campaigns regarding PCC. In order to improve the accessibility of PCC, it was suggested to simultaneously offer multiple forms: group sessions, individual consultations, walk-in-hours and online sessions, and to involve male partners. From the parental perspective, the acceptability of opportunistic offering PCC depends on the setting, context and provider characteristics.

### What this study adds to the literature

Corresponding with the findings of other studies on women’s views, our results showed that PCC is a rather unfamiliar concept [21, 25–29]. Once aware of PCC, PCC was regarded more appropriate for couples with risk factors or fertility issues. Other studies confirm that women tend not to consider themselves as a target group for PCC [8, 16, 20, 21, 25, 28, 29]. We found that parents especially appraised the potential benefit of PCC to their future child’s health, which was the most important intrinsic motivator to use PCC. A previous study on couples’ notions for preconception health found similar motivating factors for planners and interconceptional couples [22]. One of our particular findings was the perception that couples have to try to conceive for over 1 year before they are allowed to consult a healthcare provider. In accordance with the findings of a previous qualitative study by van der Zee et al., our study demonstrates that subjective norms, such as planning, privacy and medicalization may interfere with the intention to attend PCC [20]. In a study by Hosli et al. one of three most important motives for women not to respond to an invitation for PCC was perceived sufficient knowledge [8]. Although our subjects were all experienced parents, this theme did not emerge in our focus group sessions. The Internet was frequently consulted, yet having a PCC consult with a healthcare provider was still considered of added value as it allows for dialogue and suited, reliable information.

Our study reveals that, according to parents, investing in the accessibility of PCC could positively affect uptake rates. Corresponding to our findings, PCC was expected to be time-consuming in three other studies on PCC attendance [25, 27, 30]. Therefore, it was encouraged to expand on ways in which PCC is currently offered, by introducing walk-in-hours and online sessions. Previous studies have also suggested to expand access to PCC by enhancing services at community health centers and publicly-funded family planning providers [15, 31, 32]. Besides improving the accessibility, our results indicate that parents expect PCC to become more self-evident when awareness increases. Parents proposed the use of several informal channels and local settings to send promotional messages. Interestingly, raising the social dialogue and stimulating word-of-mouth marketing were regarded most efficient to make PCC a more “common” type of care, as women turn to other women for support. Evidence indeed suggests that the use of (health) services is strongly influenced by word-of-mouth and women actively seek the advice of family and friends [33]. Yet, raising the social dialogue is very difficult to attain, since it requires effort to find a way into social networks, as each community has its own specific features and needs [34].

Although the literature often recommends to engage men in PCC, our study is one of the few to consider both women’s and men’s views on PCC. A previous study by Frey et al. on the perspective of men found that although men understand the importance of optimizing their health prior to conception, this topic is hardly ever addressed by a primary care physician [23]. In our study, participating men expressed the feeling of having a neglected role when it comes to pregnancy, which was confirmed by female participants. These findings point out the need to involve men more during PCC.

Although ethnic minorities were not well represented among the study’s participants, our results indicate that social networks are more important among these groups. Therefore, we encourage the implementation of PCC prevention activities within community settings. For example, “Uma Tori”, a gender specific and culturally appropriate STI/HIV-prevention intervention, which uses interactive, multi-faceted, small-group sessions, showed a promising effect to increase awareness and improve sexual decision-making skills among women [35].

### Strengths and limitations

Strengths of this study were the amount of participants and the use of a diverse population and purposefully stratified focus groups. Although the number of participating male partners was limited, we are one of the few to have conducted a focus group session with men [22, 23]. Moreover, we used a broad approach and assessed both experiences and needs from the study population. A limitation of this study was the selection strategy, as we included participants who were already parents during the focus group sessions. This selection strategy allowed to easily identify possible participants, since they were registered at the community midwifery practice. Moreover, the experience of being a parent contributed to detailed reflections which were based on real experiences. However, due to this selection participants could have had a more positive attitude towards healthcare providers based on their experiences. Although it would have been of added value to include nulliparous couples, they are more difficult to reach, since healthcare providers are generally not aware of childbearing plans until women present after pregnancy recognition [9, 36, 37]. Therefore almost all studies concerning preconception health obtain information retrospectively, while it is surely possible with increased effort to conduct a prospective study [38]. A limitation of this research was the use of a local setting, which could potentially limit the generalizability of the results. While the population of Zeist is mostly representative for the Netherlands, educational and income levels are relatively high. Therefore, we put an effort to include participants in stratified focus groups with varying ethnic backgrounds and educational level. However, due to cancellation and no-show the study population was less diverse than was aimed at. Nevertheless, our results correspond with the findings in similar studies from other Western countries on women’s perceptions regarding PCC.

## Conclusion

Parents recommend to improve the uptake of PCC by raising awareness and accessibility. This study provides the following recommendations: (1) to promote PCC as a common type of care for every couple with a (future) child wish, stipulating that it is not necessary to wait until fertility issues arise; (2) to offer multiple complementary forms of PCC and involve men more; (3) to raise social dialogue by stimulating word-of-mouth marketing; (4) to use informal channels and local community settings for promotional messages.
